# Antipredator behaviours of a spider mite in response to cues of dangerous and harmless predators

**DOI:** 10.1007/s10493-016-0042-5

**Published:** 2016-04-11

**Authors:** Cleide Rosa Dias, Ana Maria Guimarães Bernardo, Jussara Mencalha, Caelum Woods Carvalho Freitas, Renato Almeida Sarmento, Angelo Pallini, Arne Janssen

**Affiliations:** IBED, Section Population Biology, University of Amsterdam, Science Park 904, 1098 XH Amsterdam, The Netherlands; Department of Entomology, Federal University of Viçosa, Viçosa, MG Brazil; Department of Plant Science, Federal University of Tocantins, Gurupi, TO Brazil

**Keywords:** Predation risk, *Tetranychus evansi*, *Phytoseiulus longipes*, *Phytoseiulus macropilis*, Predator–prey interaction, Trait-mediated interaction

## Abstract

Prey are known to invest in costly antipredator behaviour when perceiving cues of dangerous, but not of relatively harmless predators. Whereas most studies investigate one type of antipredator behaviour, we studied several types (changes in oviposition, in escape and avoidance behaviour) in the spider mite *Tetranychus evansi* in response to cues from two predatory mites. The predator *Phytoseiulus longipes* is considered a dangerous predator for *T. evansi*, whereas *Phytoseiulus macropilis* has a low predation rate on this prey, thus is a much less dangerous predator. Spider mite females oviposited less on leaf disc halves with predator cues than on clean disc halves, independent of the predator species. On entire leaf discs, they laid fewer eggs in the presence of cues of the dangerous predator than on clean discs, but not in the presence of cues of the harmless predator. Furthermore, the spider mites escaped more often from discs with cues of the dangerous predator than from discs without predator cues, but they did not escape more from discs with cues of the harmless predator. The spider mites did not avoid plants with conspecifics and predators. We conclude that the spider mites displayed several different antipredator responses to the same predator species, and that some of these antipredator responses were stronger with cues of dangerous predators than with cues of harmless predators.

## Introduction

Predator–prey interactions are important for population dynamics, species composition (Paine 1966) and species distributions (Sih 1980; Kats and Dill 1998; Lima 1998). Predation results in removal of prey from a population and this can affect entire ecosystems (Taylor 1984). Moreover, predators also affect the behaviour of prey through their sheer presence or through cues associated with them. The consequences of these behavioural changes can be as important as predation, affecting prey reproductive success and dynamics, species composition and ecosystem processes (Schmitz 1998; Peacor and Werner 2001; Zanette et al. 2011). Prey can respond directly to attacks by predators by trying to escape, defending themselves and by counterattacking predators. They can also perceive the presence of predators before an actual attack through cues associated with the presence of predators to subsequently avoid an encounter. Many prey can perceive chemical cues that are associated with the presence of predators and subsequently change their behaviour to reduce predation risk (Chivers and Smith 1998; Kats and Dill 1998; Peckarsky et al. 2008; Paterson et al. 2013). In response to chemical cues, prey can seek refuge (Ives and Dobson 1987; Venzon et al. 2000; Faraji et al. 2001, 2002), avoid areas with predators (Gore 1966; Brown et al. 1995; Pallini et al. 1999; Magalhaes et al. 2002; Nomikou et al. 2003; Meng et al. 2006), increase their vigilance (Sweitzer and Berger 1992), and change their habitat (Lima and Dill 1990; Bolker et al. 2003).

Besides having the obvious benefit of reducing predation risk, antipredator behaviour usually carries costs because the behavioural changes go at the expense of the time and energy spent on other activities that are important for the fitness of prey individuals (Lima 1998; Pallini et al. 1998, 1999). These costs are responsible for the non-lethal effects of predators in prey (Lima 1998). For example, trying to escape from an area in which predator cues are perceived may result in more time and energy spent on locomotion and less time spent on feeding, which may ultimately result in slower growth, reduced investment in reproduction or delayed development (Spitze 1992; Barry 1994; Ylonen and Ronkainen 1994; Koskela and Ylonen 1995; Pallini et al. 1998; Zanette et al. 2011; Lasley-Rasher and Yen 2012). Increased vigilance of parents may also result in decreased reproduction and decreased survival of offspring (Zanette et al. 2011). Another example of costs of behavioural changes in species in which juveniles run a high risk of predation is the retention of eggs and the reduced investment in egg production in prey, which obviously results in lower oviposition rates (Montserrat et al. 2007; Hua et al. 2014). To reduce costs associated with antipredator behaviour, it is important that prey can discriminate among cues from dangerous and harmless predators (Sih 1987; Venzon et al. 2000; Persons et al. 2001), investing in costly antipredator behaviour only when perceiving cues of dangerous predators. To investigate this, we studied the response of spider mites to cues of dangerous and relatively harmless predators.

In spider mites, antipredator responses to predator cues can be manifested as the avoidance of areas and plants with predators (Grostal and Dicke 1999; Pallini et al. 1999; Magalhaes et al. 2002; Choh and Takabayashi 2007), changes in oviposition site (Grostal and Dicke 1999; Lemos et al. 2010) and moving away from the leaves on which they feed to avoid leaf-dwelling predators and subsequently entering diapause to survive a period of lack of food (Kroon et al. 2008). Furthermore, several studies have shown that spider mites that perceived predator cues had a reduced oviposition rate (Oku et al. 2004; Skaloudova et al. 2007; Choh et al. 2010). Whereas most of the papers cited above concentrate on one type of antipredator response, we study all these responses in one prey spider mite species exposed to cues of harmless and dangerous predators (except for diapause induction because our study animal does not enter diapause).

Spider mites colonize host plants and subsequently increase in numbers for several generations (Helle and Sabelis 1985a). Their most important enemies are predatory mites, which invade spider mite colonies and subsequently prey and reproduce there for several generations, eventually exterminating the prey population (Helle and Sabelis 1985b; Pels and Sabelis 1999). It is therefore important that predatory mites can reproduce on the spider mites present in the colony. The spider mite used here is *Tetranychus evansi* Baker & Pritchard (Acari: Tetranychidae), a pest of tomato plants in South America, Africa and Europe (Bolland and Vala 2000; Knapp et al. 2003; Saunyama and Knapp 2003; Escudero and Ferragut 2005; Wekesa et al. 2005). It stands out among the Tetranychidae because of its high population growth rate and large temperature range (Bonato 1999) and the production of dense web (Lemos et al. 2010), which is involved in reducing predation risk (Sabelis and Bakker 1992; Lemos et al. 2010) and competition (Sarmento et al. 2011b). Furthermore, the mite suppresses tomato plant defences to levels below that in uninfested plants (Sarmento et al. 2011a). Despite its importance as pest of tomato plants, not much is known about its response to predators (Lemos et al. 2010), which is important for developing biological control strategies.

Several studies have been devoted to finding an efficient biological control agent for *T. evansi*, mainly predatory mites from the family Phytoseiidae. However, many of these report negative results, also for the predatory mite *Phytoseiulus macropilis* Banks (Acari: Phytoseidae). This predator is successfully used for biological control of the spider mite *Tetranychus urticae* Koch, a species closely related to *T. evansi* (Watanabe et al. 1994; de Moraes et al. 2004; Oliveira et al. 2007, 2008, 2009). It can feed on all stages of prey, but eggs and young instars are more vulnerable (A. Janssen and F. Lemos, pers. obs.). However, *P. macropilis* has a low predation rate and cannot complete its life cycle when feeding on *T. evansi* and is not associated with it in the field (de Moraes and McMurtry 1985; Rosa et al. 2015). We therefore characterize it as a harmless predator for *T. evansi*.

In contrast, the closely related predatory mite *Phytoseiulus longipes* Evans (Acari: Phytoseidae) has a high population growth rate and high predation rate when feeding on *T. evansi* (de Moraes and McMurtry 1985). This predatory mite is also associated with Tetranychid populations in the field, it can develop and reproduce well when feeding on *T. urticae* or *T. evansi* (Ferrero et al. 2007; Furtado et al. 2007a), and is being considered as candidate for biological control of *T. evansi* populations on tomato (Furtado et al. 2007b; Silva et al. 2010; Ferrero et al. 2011). It can feed on all development stages of *T. evansi*, but eggs and young instars are most vulnerable. We therefore considered *P. longipes* as a dangerous predator for *T. evansi*.

We investigated the response of *T. evansi* to predator cues. The predators could not encounter the prey, but prey could perceive cues associated with predators. The objective of this study was to investigate whether *T. evansi* responded more strongly to cues of the dangerous predator *P. longipes* than to cues of the relatively harmless *P. macropilis*. We studied changes in oviposition behaviour of the spider mites to cues left behind by the predators, we assessed their escape behaviour in response to predator cues, and we studied their avoidance of plants with predator cues. In all cases, we expected that cues of dangerous predators would result in stronger changes of the behaviour than cues of harmless predators would.

## Materials and methods

### Rearing methods

Tomato plants (*Solanum lycopersicum*, variety Santa Clara I-5300) were grown in pots (2 L) using a commercial substrate, composed of vermiculite plus organic fertilizer. The plants were kept inside a greenhouse and fertilized with NPK (4-14-8) and superphosphate. The plants were watered according to their needs.

*Tetranychus evansi* was obtained in 2002 at the campus of the Federal University of Viçosa, Brazil, from naturally infested tomato plants of the same variety as above. Spider mites were cultured on tomato leaves with the petioles in a plastic tube with water to maintain leaf turgidity. The tubes were kept in plastic trays filled with detergent and water, which served to prevent mite escapes and invasion of other arthropods. A clean tomato leaf was added to the *T. evansi* culture every 2 days.

The predatory mite *P. longipes* was kindly provided by Professor Gilberto de Moraes (University of São Paulo, Brazil) in 2006. The predatory mite *P. macropilis* was found to spontaneously invade bean plants infested by *T. urticae* in a greenhouse on the campus mentioned above. Both *P. longipes* and *P. macropilis* were cultured in similar rearing units as the spider mites, but were supplied with leaves infested by spider mites. As explained above, *P. macropilis* cannot be reared on *T. evansi*, so it was fed *T. urticae*; the culture of *P. longipes* was fed *T. evansi*. Both predatory mite cultures were maintained under controlled conditions in a room (25 ± 2 °C, 80 ± 10 % relative humidity and 12 h light).

### Oviposition in the presence of predator cues

Leaf discs (Ø = 2.4 cm) were cut from tomato leaves, with the central vein in the middle of the discs. They were placed individually in a Petri dish (Ø = 4.0 cm) on top of a hydrophilic sponge soaked in water, which served to maintain the turgidity of the leaf disc. A thin line of hydrophilic cotton wool was placed on the vein, touching the water at both ends. Predatory mites cannot cross such humid cotton wool barriers (Pallini et al. 1998). Twenty-five adult female predators, either *P. longipes* or *P. macropilis* were placed on one of the disc halves; the other half remained clean. The predators and their eggs were carefully removed after 4 h. Hence, one disc half contained cues consisting of faeces, exuviae and chemical cues left by the predatory mites. These cues could be easily perceived by the spider mites because they were concentrated due to the high density of predators used. Subsequently, the cotton wool was removed and the vein was dried with tissue paper. One adult female spider mite, aged 12 days since egg, was released in the centre of each disc. Oviposition on each side of the disc was evaluated 24 h after release, and was analysed using a generalized linear model (GLM) with a quasi-Poisson error distribution. The effect of the cues of the two predator species on the distribution of eggs over disc halves was compared with a GLM with a quasi-binomial error distribution. Because spider mites are known to produce fewer eggs as a response to predator cues (Oku et al. 2004; Skaloudova et al. 2007; Choh et al. 2010), we also offered leaf discs entirely covered with predator cues or entirely clean. Discs of the same size as above either received 25 female predators for 4 h or were left clean. The same number of both species of predatory mites was used here as above, hence, the same amount of cues was present but on a twice as large a surface. The predators and their eggs were removed as above and one adult female spider mite was again released at the centre of each disc. The oviposition rate was measured as above. Twenty replicates were done per predator species. Oviposition rates were analysed using a GLM with Poisson error distribution with the presence of predator cues and predator species as factors.

### Escapes from leaf discs with predator cues

In the above experiments, spider mites could only escape by entering the water surrounding the leaf discs, which they are somewhat reluctant to do. To evaluate escapes by *T. evansi*, we therefore prepared another type of arena, consisting of two discs (Ø = 2.5 cm) connected by a bridge (1.5 × 1.0 cm), cut as one piece from a single tomato leaflet. A thin line of hydrophilic cotton wool was placed on the connection of the bridge with the two discs to restrain mites on each disc. Ten females of *T. evansi* were released on both discs and 25 adult females of one of the two predator species were released on one of the discs 24 h later. Four hours after releasing the predators, the herbivores, predators and the cotton wool were removed from the discs and the arena was dried. Hence, these discs contained cues of prey, predators and cues associated with predation, in contrast to the previous experiments, in which only cues of predators were present. One female of *T. evansi* was released at the centre of the disc with *T. evansi* and predator cues, from where she could escape to the disc with only *T. evansi* cues. As control, one female of *T. evansi* was released on one of two connected discs, both only with *T. evansi* cues. The position of the female was evaluated after 48 h. Twenty replicates were done per treatment per predator species. The incidence of spider mites that had left the release disc after 48 h was analysed with a GLM with a binomial (*P. longipes*) and quasi-binomial (*P. macropilis*) error distribution.

### Avoidance of plants with predator cues

The three highest leaves of six tomato plants with four completely developed leaves were infested with *T. evansi*, around 400 spider mites per plant, during 7 days. To prevent spider mites from moving from the upper leaves to the lowest leaf, a ring of glue was applied to the main stem of the plants immediately above the first leaf, isolating it from the rest of the plant (Pallini et al. 1999). Six days after infestation, the plants were allocated equidistantly in a hexagon (Ø = 80 cm) in a cage consisting of a frame (160 × 160 × 120 m) covered with thin screen and a tarpaulin bottom (160 × 160 × 15 cm) filled with soil. The pots containing the plants were buried and soil was added to the pots so that soil levels inside and outside the pots were equal (Pallini et al. 1998; Janssen 1999; see Nomikou et al. 2003 for a drawing of the set-up). The cage was situated outside, in an area shielded from extreme wind by a building on one side and a steep slope opposite, thus ensuring that the mites could not reach the plants through dispersal on air currents, but only by walking. To provide cues of predation risk, 100 adult females of *P. longipes* or *P. macropilis* were added to the three highest leaves with spider mites of three of the plants. Plants without predators were alternated with plants with predators in the hexagon. To control for any unforeseen directionality in the searching behaviour of the mites, care was taken that each plant position was occupied by plants without predators in half of the replicates, and by plants with predators in the other half (Janssen 1999). Hence, there were two configurations, one with plants with predators on positions 1, 3, and 5, and one with plants with predators on positions 2, 4, and 6.

Two hundred adult female spider mites were collected in a Petri dish, which was placed in the middle of the hexagon 24 h after the predators were added to the plants. The spider mites could disperse freely by walking over the soil to the plants. The stem of tomato plants contains high densities of trichomes, which impede movement of mites. Therefore, a wooden stick was inserted into the soil near the base, touching the lowest leaf, forming a bridge from the soil surface to the first leaf, giving the mites access to the leaf. Because of the glue barrier, the spider mites could not reach the leaves with predators, so they could only perceive volatile cues associated with predators. During the first 6 h and subsequently after 24 and 30 h, the mites that arrived on the lowest leaf of the plants were counted and removed.

To verify whether the set-up was suitable for assessing preference of *T. evansi*, we made use of the preference of this mite for plants with conspecifics over clean plants (Sarmento et al. 2011a). Three plants infested by *T. evansi* interspersed with three clean plants were placed in the same hexagon and spider mites were released at the center.

All experiments were replicated four times with four different sets of plants and different groups of mites. Square-root transformed numbers of mites recaptured on the plants were analysed using a linear mixed effects model (LME of the nlme package in R, R Development Core Team 2013). Replicates were used as random factor and the treatments of plants (with or without predators), time and the configuration of plants within the hexagon as factors.

## Results

### Oviposition in the presence of predator cues

**Fig. 1:**
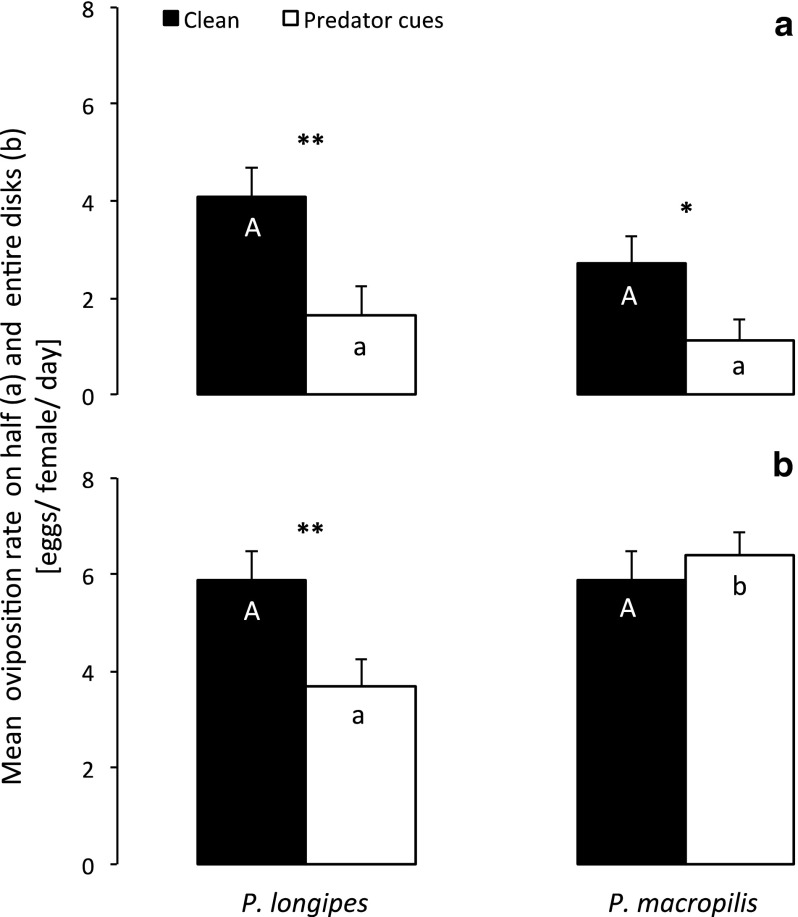
Mean (+SE, n = 20 females) oviposition of *Tetranychus evansi* after 24 h. **a** One half of each leaf disc had cues of predators whereas the other half did not. **b** Discs were either completely covered with cues of *Phytoseiulus longipes* or *P. macropilis*, or without such cues. *Black bars* indicate oviposition on discs (**b**) or disc halves (**a**) without predator cues, *white bars* that on discs (**b**) or disc halves (**a**) with predator cues. *Asterisks* indicate significant differences between treatment and control for each predator species separately; *different letters* indicate a significant difference between treatments with the different predator species, but for the same treatment, i.e. comparing the two *white bars* or the two *black bars* (GLM, *p* < 0.05)

Spider mites laid more eggs on the clean disc halves than on halves with cues of *P. longipes* (GLM: F_1,38_ = 7.66, *p* = 0.0086) or of *P. macropilis* (F_1,34_ = 4.54, *p* = 0.04) (Fig. 1a). When comparing the response of *T. evansi* to both predator species, there was no significant difference in oviposition behaviour (GLM: F_1,36_ < 0.001, *p* = 0.98; Fig. 1a). With cues of *P. longipes*, females produced on average 4.1 (SE = 0.57) eggs on the clean disc half and 1.65 (SE = 0.57) on the half with cues. With cues of *P. macropilis*, they produced 2.72 (SE = 0.57) eggs on the clean half and 1.11 (SE = 0.46) on the half with predator cues.

When entire discs had cues of predators, *T. evansi* laid significantly fewer eggs on the disc with cues of *P. longipes* than on clean discs (GLM: Deviance = 10.05, *df* = 1,37, *p* = 0.0015; Fig. 1b). In contrast, the spider mites showed no significant difference in oviposition on the discs with or without *P. macropilis* cues (GLM: Deviance = 0.35, *df* = 1,33, *p* = 0.55; Fig. 1b). When comparing the response to both predators, *T. evansi* laid fewer eggs on discs with cues of *P. longipes* than with *P. macropilis* cues (GLM: Deviance = 12.5, *df* = 1,38, *p* = 0.0004; Fig. 1b). Oviposition on clean discs did not differ (i.e. the controls of the experiments with *P. longipes* and *P. macropilis* cues, GLM: Deviance < 0.001, *df* = 1,38, *p* = 1; Fig. 1b).

### Escapes from leaf discs with predator cues

**Fig. 2:**
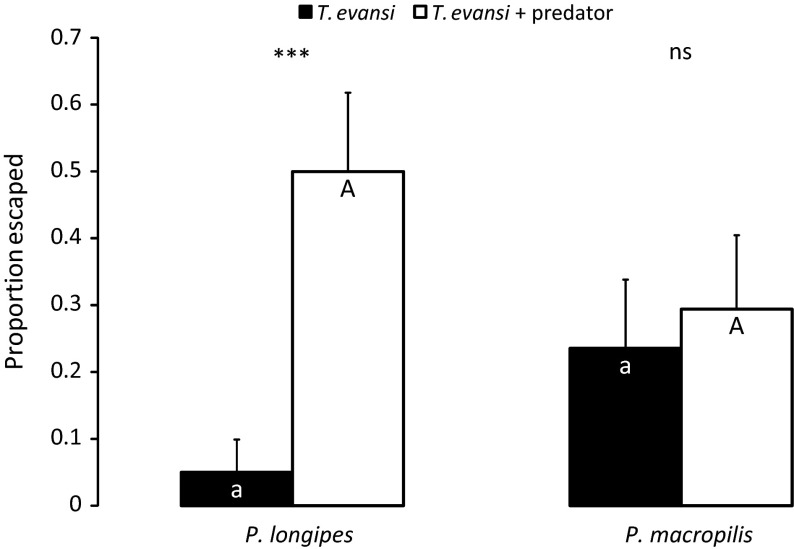
Proportion (+SE, n = 20 females) of *Tetranychus evansi* that escaped from the arena where they were released. *Black bars* indicate the proportion of mites that escaped from discs without predator cues, *white bars* indicate that from discs with predator cues (*Phytoseiulus longipes* or *P. macropilis*). *Asterisks* indicate significant differences between treatment and control for each predator species separately; *different letters* indicate a significant difference between treatments with the different predator species, but for the same treatment, i.e. comparing the two *white bars* or the two *black bars* (GLM, *p* < 0.05)

*Tetranychus evansi* escaped significantly more from discs with cues of *P. longipes* to a connected disc without predator cues than from discs without predator cues (GLM: Deviance = 10.9, *df* = 1,36, *p* = 0.001; Fig. 2). This did not hold for spider mites experiencing cues of *P. macropilis* (GLM: Deviance = 0.15, *df* = 1,32, *p* = 0.70; Fig. 2). When comparing the escapes between experiments, the proportion of escapes from discs with cues of only prey only did not differ significantly (cf. dark bars of Fig. 2, GLM: Deviance = 2.81, *df* = 1,35, *p* = 0.093), neither did the numbers of escapes from discs with predator cues (cf. light bars of Fig. 2, Deviance = 1.56, *df* = 1,33, *p* = 0.21).

### Avoidance of plants with predator cues

**Fig. 3:**
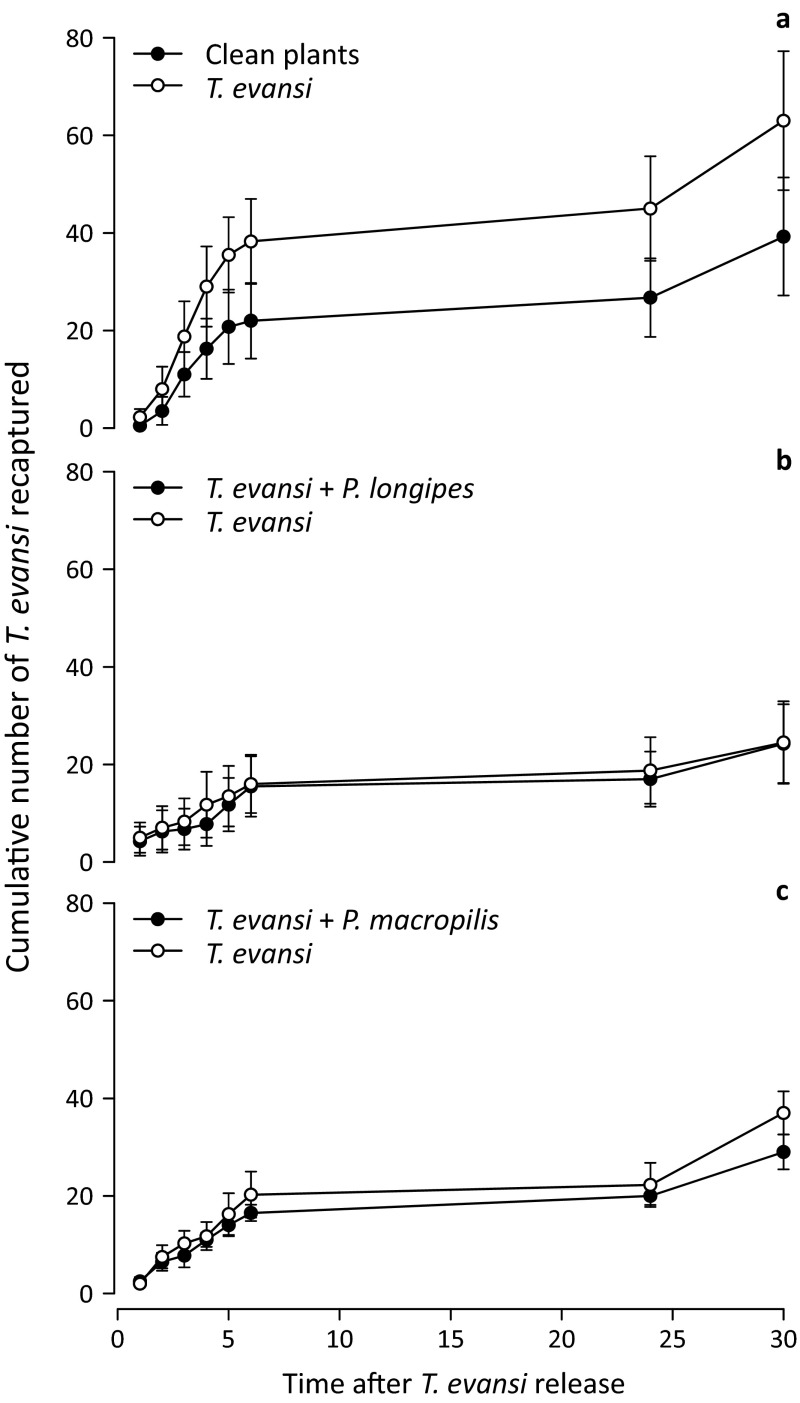
Cumulative number (±SE) of *Tetranychus evansi* recaptured per plant through time on plants. **a** Plants either contained *T. evansi* (*empty circles*) or were clean (*filled circles*); **b** plants either contained *T. evansi* (*empty circles*) or *T. evansi* plus the predatory mite *Phytoseiulus longipes* (*filled circles*); **c** plants either contained *T. evansi* (*empty circles*) or *T. evansi* plus the predatory mite *P. macropilis* (*filled circles*)

Spider mites were recaptured more often on plants infested with conspecifics than on clean plants (LME, Likelihood ratio = 9.87, *df* = 4, *p* = 0.0017; Fig. 3a) and there was a significant effect of time on the numbers of mites recaptured (LME, Likelihood ratio = 30.2, *df* = 4, *p* < 0.0001). These results show that the mites can discriminate between plants that received different treatments in the set-up used.

There was no significant difference in the numbers of *T. evansi* recaptured on plants with or without *P. longipes* or *P. macropilis* (Fig. 3c, LME, Likelihood ratio = 0.48, *df* = 5, *p* = 0.48; Fig. 3b, Likelihood ratio = 0.16, *df* = 6, *p* = 0.68, respectively). There was a significant effect of time (LME, Likelihood ratio = 7.06, *df* = 5, *p* = 0.0079, Fig. 3) and position (Likelihood ratio = 5.38, *df* = 5, *p* = 0.02) on the numbers of mites recaptured in the experiment with *P. longipes* cues. For unknown reasons, more spider mites were recaptured on all plants when plants with *P. longipes* were on positions 1, 3, and 5 than when plants with these predators were on positions 2, 4, and 6. In the experiments with *P. macropilis*, there was a significant effect of the interaction between time and configuration (LME, Likelihood ratio = 6.03, *df* = 5, *p* = 0.014) on the numbers of mites recaptured. This was caused by an increase in the numbers of spider mites recaptured on plants without predators after 30 h, but only in one of the two configurations (data not shown). The reason for this increase is unknown so far. Nevertheless, the overall numbers of spider mites recaptured on plants with or without *P. macropilis* did not differ significantly. Comparison of the total percentage of spider mites recaptured in the three release experiments showed that *T. evansi* was recaptured more often when it was offered plants infested with *T. evansi* and clean plants (51.1 %) than when it was offered plants infested with *T. evansi* and predatory mites (*P. longipes* 24.3 % and *P. macropilis* 33.0 %) (GLM, F_2,10_ = 13.2, *p* < 0.001, Fig. 3).

## Discussion

Many prey evolved the capacity to detect cues associated with predators and change their behaviour in response to those cues, even in the absence of predators (Chivers and Smith 1998; Kats and Dill 1998). We show here that the spider mite *T. evansi* changes its behaviour in response to cues of dangerous and harmless predators. Moreover, we found that this spider mite shows various types of behavioural changes to cues of the same predator. We found partial support for the expectation that the spider mites would show a stronger response to cues of the dangerous predator than to cues of the harmless predator. Specifically, we assessed three responses that were studied separately in spider mites thus far, i.e. their oviposition behaviour, escape behaviour and avoidance. First, the spider mites showed changes in oviposition behaviour, laying eggs more often on leaf disc halves without predator cues than on halves with predator cues. Contrary to expectation, the response to cues of the harmless predator did not differ from that to cues of the dangerous predator (Fig. 1a). A possible explanation for this is that the spider mites could move from one leaf disc half to the other within a few minutes; hence, they did not loose much time or energy because of this antipredator behaviour. Possibly, it then pays to even respond to cues of relatively harmless predators.

Furthermore, the spider mites produced fewer eggs on leaf discs entirely covered with predator cues than on clean leaf discs. The response to cues of the dangerous predator was stronger than that to cues of the harmless predator (Fig. 1b), which can be explained by the costs involved in this antipredator behaviour; reduced oviposition results in reduced offspring production. This reduced oviposition might have been caused by prey retaining their eggs to avoid predation (Montserrat et al. 2007). Alternatively, the spider mites may have tried to escape from the leaf discs, thus spending less time feeding. Because feeding is strongly related to egg production in spider mites (Sabelis 1991), this will also have resulted in lower oviposition rates. Further experiments should be done to clarify whether the reduced oviposition rate was caused by egg retention, by reduced feeding, or by both (Montserrat et al. 2007).

Second, the spider mites escaped more frequently from leaf discs with cues of the dangerous predator than from clean discs (Fig. 2), but not from discs with cues of the harmless predator (Fig. 2), again showing that the response to cues of the dangerous predator was stronger that that to cues of the harmless predator. In this case, escaping to the leaf disc with predator cues would involve more time than walking from one leaf disc half to another, as above. The spider mites had to find the bridge connecting two leaf discs and had to cover a larger distance to arrive at the other leaf disc.

Third, spider mites did not avoid plants with predators: similar numbers were recaptured on plants with predators as on plants without predators (Fig. 3b, c). It could be argued that the predation risk of individual mites on a plant with hundreds of adult spider mites and numerous eggs and immatures is rather low, thus, not avoiding plants with predators would not increase predation risk very much. However, earlier experiments using the same experimental set-up showed that a closely related spider mite species did avoid going to plants with one species of predator, but not to plants with another predator species (Pallini et al. 1999). We recaptured more spider mites when they were offered plants infested with *T. evansi* and clean plants than when offered plants infested with *T. evansi* and predatory mites (cf. Figure 3a with 3b, c). Hence, in the presence of cues from predators, fewer spider mites were found back on any plant (including plants without predators). Possibly, the spider mites tried to move away from plants with predators and thus spent more time walking before reaching a plant, or they chose to disperse away from the entire experimental arena in response to cues associated with predators. If so, the spider mites did also show antipredator behaviour in this experiment. In any case, they did not respond differently to the two predator species.

The cues perceived by the spider mites differed among experiments. Whereas the cues present in the experiments with the single leaf discs originated from the predators (feces, exuviae), the experiment with the connected discs also involved cues associated with predation, such as remains of dead conspecifics and perhaps prey alarm pheromones (Brown et al. 1995; Janssen et al. 1997; Grostal and Dicke 1999; Chivers and Smith 1998). Predation also occurred in the release-recapture experiment, but in this experiment, the spider mites could only perceive volatile cues, associated with conspecifics and with predation. Experiments with closely related spider mites and predators have demonstrated the existence of such volatile cues (Janssen et al. 1997; Pallini et al. 1999).

The diet of predators is known to affect antipredator behaviour: prey show stronger antipredator behaviour when perceiving cues from predators that recently fed on the same prey species than when perceiving predators that fed on other prey species (Venzon et al. 2000; Persons et al. 2001; van Maanen et al. 2015). Here, the predatory mite *P. longipes* was reared with *T. evansi* as prey and *P. macropilis* was reared on *T. urticae*. It is possible that *T. evansi* showed a weaker change in oviposition behaviour to *P. macropilis* because it had previously fed on another prey. This does not hold for the experiments on escape and avoidance behaviour, where both predator species had been feeding on *T. evansi* for some time.

In conclusion, our results show that the prey changed their behaviour when perceiving predator cues, but their response to cues associated with dangerous predators was not always stronger than to cues associated with harmless predators. In general, it is expected that prey should discriminate between cues from dangerous and harmless predators when the costs of antipredator behaviour are high (Sih 1982; 1987; Venzon et al. 2000; Persons et al. 2001). The lack of difference in response to dangerous and harmless predators found in some experiments here may partly have been caused by the low costs involved in antipredator behaviour, such as moving for a short time and over short distances (Sih 1982). Although many studies have addressed costs and benefits of antipredator behaviour, they have often considered only one type of behaviour. Here we show that prey show various types of antipredator behaviour to cues associated with the same predator species. We suggest that the various options of antipredator behaviour open to prey should all be considered, and their relative costs and benefits should be assessed to predict the most adequate type of response under various circumstances.
